# The presence of nitrate dramatically changed the predominant microbial community in perchlorate degrading cultures under saline conditions

**DOI:** 10.1186/s12866-014-0225-3

**Published:** 2014-09-07

**Authors:** Victor G Stepanov, Yeyuan Xiao, Quyen Tran, Mark Rojas, Richard C Willson, Yuriy Fofanov, George E Fox, Deborah J Roberts

**Affiliations:** School of Engineering, University of British Columbia, EME 4261, 3333 University Way, Kelowna, BC Canada; Department Biology & Biochemistry, University of Houston, Houston, TX 77204-5001 USA; Department Computer Sciences, University of Houston, Houston, TX 77204-3010 USA; Department Chemical & Biomolecular Engineering, University of Houston, Houston, TX 77204-4004 USA; Centro de Biotecnología FEMSA, Departamento de Biotecnología e Ingeniería de Alimentos, Tecnológico de Monterrey, Monterrey, 64849 Mexico

**Keywords:** Perchlorate contamination, Nitrate, Metagenomic sequencing, Community analysis, Salt-tolerance

## Abstract

**Background:**

Perchlorate contamination has been detected in both ground water and drinking water. An attractive treatment option is the use of ion-exchange to remove and concentrate perchlorate in brine. Biological treatment can subsequently remove the perchlorate from the brine. When nitrate is present, it will also be concentrated in the brine and must also be removed by biological treatment. The primary objective was to obtain an in-depth characterization of the microbial populations of two salt-tolerant cultures each of which is capable of metabolizing perchlorate. The cultures were derived from a single ancestral culture and have been maintained in the laboratory for more than 10 years. One culture was fed perchlorate only, while the other was fed both perchlorate and nitrate.

**Results:**

A metagenomic characterization was performed using Illumina DNA sequencing technology, and the 16S rDNA of several pure strains isolated from the mixed cultures were sequenced. In the absence of nitrate, members of the *Rhodobacteraceae* constituted the prevailing taxonomic group. Second in abundance were the *Rhodocyclaceae*. In the nitrate fed culture, the *Rhodobacteraceae* are essentially absent. They are replaced by a major expansion of the *Rhodocyclaceae* and the emergence of the *Alteromonadaceae* as a significant community member. Gene sequences exhibiting significant homology to known perchlorate and nitrate reduction enzymes were found in both cultures.

**Conclusions:**

The structure of the two microbial ecosystems of interest has been established and some representative strains obtained in pure culture. The results illustrate that under favorable conditions a group of organisms can readily dominate an ecosystem and yet be effectively eliminated when their advantage is lost. Almost all known perchlorate-reducing organisms can also effectively reduce nitrate. This is certainly not the case for the *Rhodobacteraceae* that were found to dominate in the absence of nitrate, but effectively disappeared in its presence. This study is significant in that it reveals the existence of a novel group of organisms that play a role in the reduction of perchlorate under saline conditions. These *Rhodobacteraceae* especially, as well as other organisms present in these communities may be a promising source of unique salt-tolerant enzymes for perchlorate reduction.

**Electronic supplementary material:**

The online version of this article (doi:10.1186/s12866-014-0225-3) contains supplementary material, which is available to authorized users.

## Background

The perchlorate ion has been found to contaminate ground and surface water, as well as food, vitamins, and drinking water [1-5]. Perchlorate is known to inhibit the sodium-iodide symporter, which is responsible for the supply of iodine to the thyroid and to milk in the mammary glands [6].

The environmentally stable perchlorate ion can be removed from contaminated water using ion-exchange in combination with a microbial treatment processes. During bacterial respiration perchlorate acts as an electron acceptor and requires the addition of an electron donor [7-9]. Perchlorate is metabolized through a series of reductions to chlorite and then a dismutation to chloride and molecular oxygen [10]. In many cases, perchlorate is present in very low concentrations and must be treated to even lower levels, e.g., 6 ppb in California [11], and 2 ppb in Massachusetts [12]. This makes biological treatment very difficult as a stand-alone process since other electron acceptors such as nitrate and oxygen are often preferred.

Ion-exchange processes have been used to remove perchlorate to meet the required treatment goals, but the perchlorate and other co-contaminant anions such as nitrate are merely transferred to a concentrated brine stream, which in turn must be treated [13,14]. The combination of ion-exchange to remove and concentrate the perchlorate from the contaminated water source and biological treatment to remove the perchlorate from the brine is an attractive option for drinking water treatment. Since nitrate is often present with perchlorate and will also be concentrated in the ion-exchange brine, the biological system should also remove nitrate.

The cultures examined here were initially enriched from marine sediment in the year 2001. The culture designated as P30 has been maintained continuously in the laboratory in a defined 3% NaCl saline medium with perchlorate and acetate as the sole electron acceptor and donor respectively in a 1.5 L batch reactor [15]. In 2003, half of the P30 culture was transferred to a new reactor and fed with both nitrate and perchlorate as the electron acceptors and acetate as the sole electron donor. This culture was designated as NP30. The cultures have been used as the basis for many published studies over the last 10 years [13,15-23].

In order to better understand these cultures, an initial characterization of the microbial populations was obtained by cloning 16S rDNA genes and characterizing them by either DGGE or 16S rDNA sequencing [21]. In the work reported here, we further our understanding of the biota in these cultures by characterizing the total community DNA of both the P30 and NP30 cultures using Illumina DNA sequencing technology. This metagenome analysis is supplemented by sequencing the 16S rRNA genes of several strains that have been isolated from the cultures and successfully cultivated in the laboratory. The combination of these two approaches provides new insight into the major and minor components of the cultures and their contribution to perchlorate and nitrate removal from contaminated water.

## Results

### Pure culture isolation

Four pure strains were obtained using anaerobic incubations: NW (white colonies) and NY (yellow colonies), originated from NP30; PW (white colonies) and PY (yellow colonies), originated from P30. Another four pure strains were obtained from aerobically incubated plates and designated as NWO, NYO, PWO and PYO following the naming scheme for anaerobic isolates. When these new strains were transferred back into P medium, none of them could reduce perchlorate after 120 days’ incubation, although growth was observed. A single additional isolate that can reduce perchlorate was obtained from the NP30 culture and characterized in detail as reported elsewhere [24]. This strain was identified as *Marinobacter* sp. strain P4B1 a member of the *Alteromonadaceae*. 16S rDNA sequences were obtained for all the pure strains except the NW isolate, which produced heterogeneous sequencing patterns. Table 1 presents a summary of the results obtained by comparing the sequences obtained for the pure cultures with those from the NCBI *nt* database. All belong to the phylum Proteobacteria. Two of the NP30 strains were identified as members of the *Rhodobacteraceae* closely related to *Stappia indica*. The other was an *Azoarcus* like isolate close to the members of a recently discovered and vaguely defined genus *Denitromonas* (*Rhodocyclaceae*). Three of the five P30 isolates were identified as *Hyphomonas jannaschiana.* The remaining P30 isolate was identified as a *Cellulomonas* strain that belongs to the *Cellulomonadaceae* and is closely related to the *C. hominis* type strain.

**Table 1:** **Sequencing summary for 16S rDNA from pure cultures**

**ID**	**Origin**	**Accession number**	**Length (bp)**	**Closest BLAST match in NCBI** ***nt*** database*)	**Identity (%)**	**Bit score**	**Taxonomic affiliation**	
				**(excluding uncultured/environmental samples)**			**Family**	**Genus & Species**
NWO	NP30	KF135667	1340	*Stappia indica* str. R32 (AB607882)	99	2329	*Rhodobacteraceae*	*Stappia indica*
NY	NP30	KF135668	1379	*Denitromonas indolicum* str. MPKc (AY972852), *Denitromonas aromaticu*s (AB049763)	95	2183	*Rhodocyclaceae*	*Denitromonas* sp.*)
NYO	NP30	KF135669	1343	*Stappia indica* str. R32 (AB607882)	99	2334	*Rhodobacteraceae*	*Stappia indica*
PW	P30	KF135670	1307	*Hyphomonas jannaschiana* W6-15 (DQ659446)	99	2331	*Hyphomonadaceae*	*Hyphomonas jannaschiana*
PWO	P30	KF135671	1297	*Hyphomonas jannaschiana* W6-15 (DQ659446)	99	2313	*Hyphomonadaceae*	*Hyphomonas jannaschiana*
PY	P30	KF135672	1373	*Cellulomonas* sp. SJH-002 (KC335136)	99	2435	*Cellulomonadaceae*	*Cellulomonas* sp.
PYO	P30	KF135673	1297	*Hyphomonas jannaschiana* W6-15 (DQ659446)	99	2313	*Hyphomonadaceae*	*Hyphomonas jannaschiana*
P4B1	NP30	JN861074	1502	*Marinobacter* sp. SCSWE03 (FJ461458)	99	2513	*Alteromonadaceae*	*Marinobacter* sp.

*)“*Denitromonas*” is not a validly published genus; the closest properly defined genus is *Azoarcus.*

### Comparative metagenomic analysis

**Figure 1:**
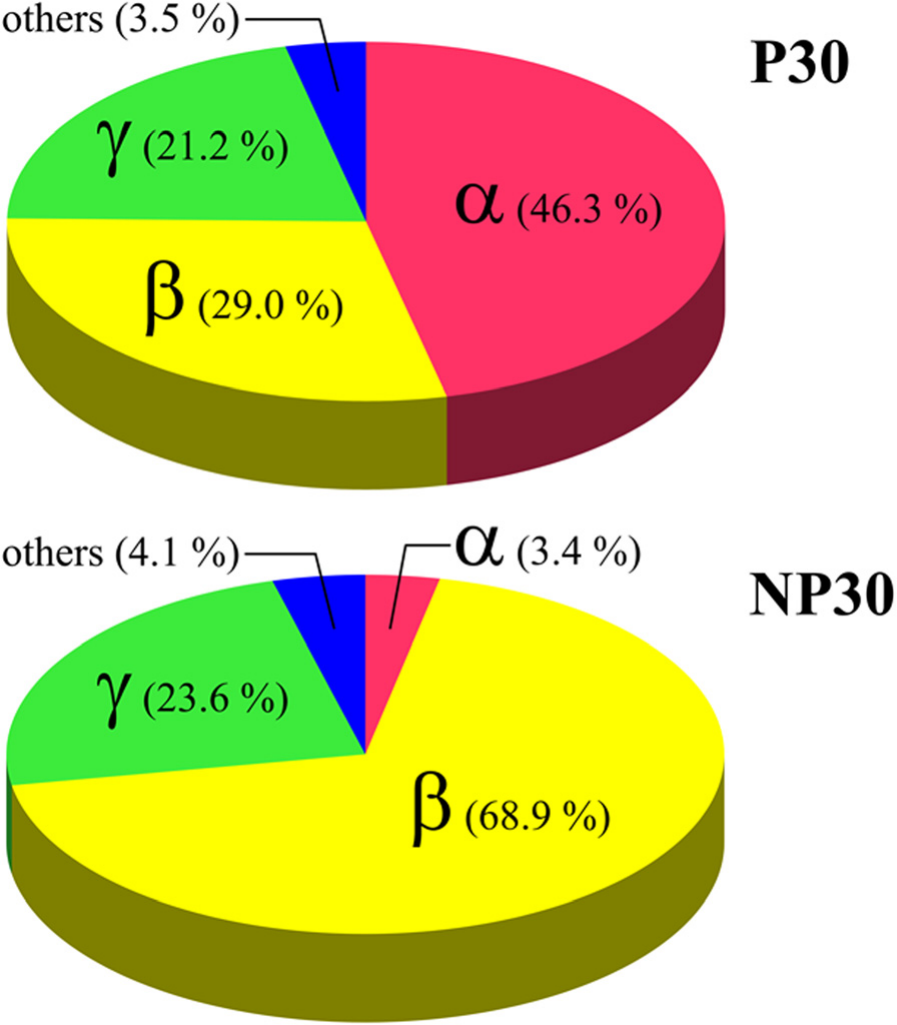
**The relative abundance of members of the Alpha, Beta, and Gamma Proteobacter are shown in a pie chart with approximate percentages indicated.** The dramatic decrease of the Alphaproteobacter in the NP30 culture and accompanying increase in the Betaproteobacter is clearly illustrated.

**Figure 2:**
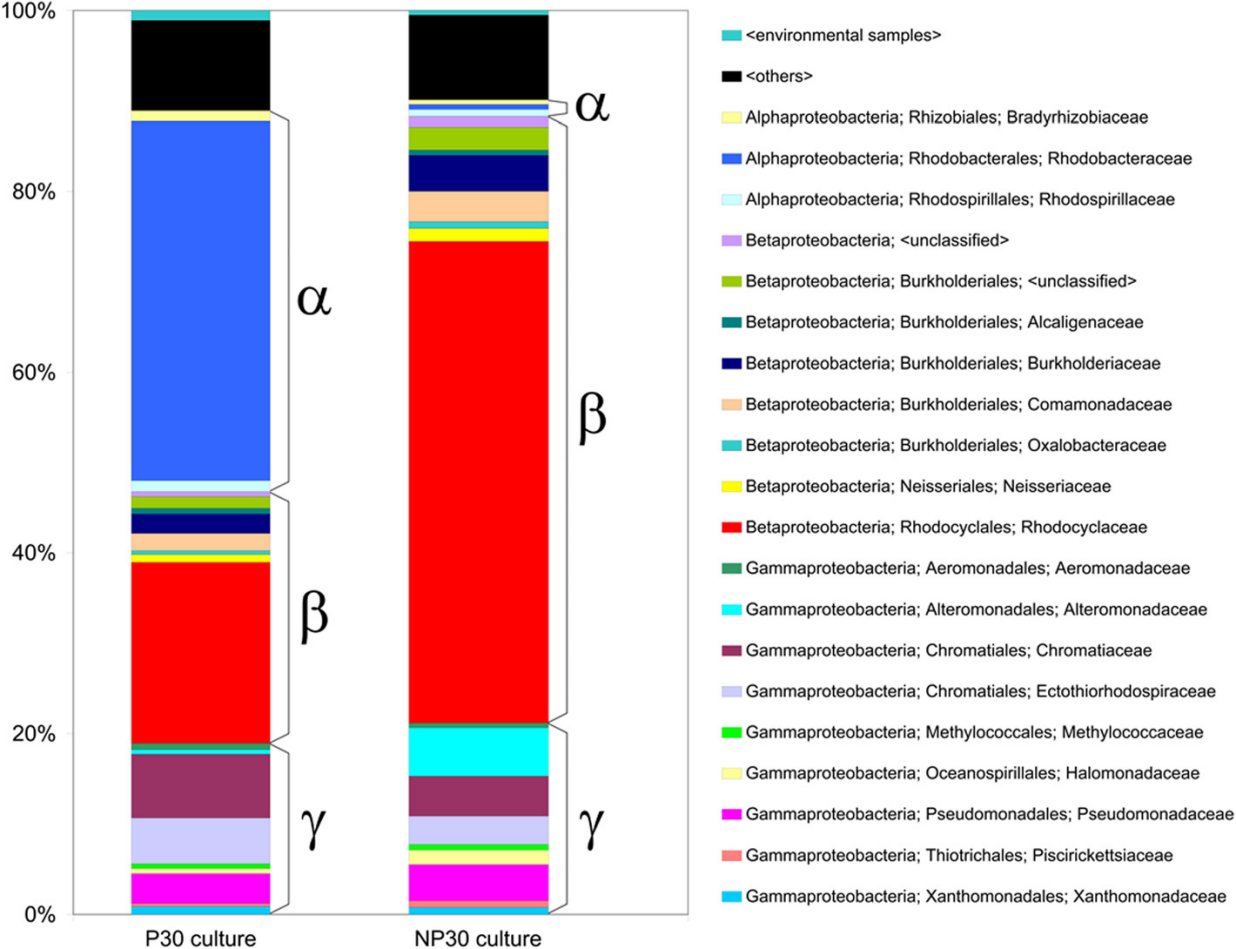
**Relative abundance of major bacterial families in P30 and NP30 communities.** Relative abundance was estimated using the ContigEval analysis pipeline. Height of the colored boxes represents a number of identities in all contigs assigned to a given taxon related to a number of identities in all contigs assigned to all taxa. The boxes are clustered by class. Names associated with each color are provided that list Class, Order, and Family. For each culture, only the most abundant families contributing to the 90% of cumulative percentage are shown. An alternative representation in which they are ordered by taxon abundance averaged for P30 and NP30 cultures is provided as Additional file 4.

Illumina sequencing of the two cultures yielded approximately 7.5 million reads for P30 and 6.3 million for NP30. Table 2 summarizes the assembly of the metagenomic contigs. The data analysis strategy yielded converging estimates for the taxonomic composition of the P30 and NP30 microbial communities at the family level and above (Additional files 1, 2 and 3). As shown in Figure 1, both mixed cultures were found to be dominated by Alpha, Beta, and Gamma Proteobacteria, while the proportion of other phyla is substantially lower. As shown in Figure 2, Alphaproteobacteria from the family *Rhodobacteraceae* constitute a prevailing taxonomic group in the P30 culture (Figure 2, Additional file 4). The second most abundant fraction is formed by Betaproteobacteria belonging to *Rhodocyclaceae*, which is followed by Gammaproteobacteria from families *Chromatiaceae*, *Ectothiorhodospiraceae* and *Pseudomonadaceae*. In the NP30 culture, Figure 2, the *Rhodobacteraceae* have effectively completely disappeared and are now replaced by an expanding representation from the *Rhodocyclaceae* and the emergence of the *Alteromonadaceae* at a much lower but increasingly significant fraction of the community. Other taxonomic groups found in the NP30 community include Gammaproteobacteria from the families *Pseudomonadaceae*, *Ectothiorhodospiraceae* and *Chromatiaceae* and Betaproteobacteria from families *Burkholderiaceae* and *Comamonadaceae*.

**Table 2:** **Summary of metagenomic contig assembly**

	**P30 culture**	**NP30 culture**
Number of 36-mer reads collected	7,551,046	6,307,535
Number of assembled contigs	42,437	27,725
Number of nucleotides in contigs > 100 nt	9,850,121	5,030,930
Longest sequence, nt	3,421	5,099
Mean, nt	232	181
N50, nt	258	183
Shortest sequence, nt	100	100

Estimates of the P30 culture composition at the genus level vary depending on the employed data processing procedure. When the contigs were evaluated against NCBI *nt* nucleotide database (MEGAN, ContigEval), the highest scoring taxon was the genus *Ruegeria* (*Rhodobacteraceae*). However, when contig-derived protein sequences were searched against protein databases, the major portion of the hits was assigned to other members of the same family, either *Roseovarius* or *Roseobacter*. This discrepancy may reflect an influence of non-coding genomic sequences or codon usage pattern on the taxonomic assignment of the contigs. Other highly represented genera included *Rhodobacter*, *Dinoroseobacter* and *Phaeobacter* (*Rhodobacteraceae*), *Azoarcus* and *Thauera* (*Rhodocyclaceae*), *Burkholderia* (*Burkholderiaceae*), *Pseudomonas* (*Pseudomonadaceae*), *Allochromatium* (*Chromatiaceae*) and *Thioalkalivibrio* (*Ectothiorhodospiraceae*). While the abundance rank of the mentioned taxons depends on the contig binning method used, they can often be found among the top genera. The composition of the NP30 culture exhibits less ambiguity. The most abundant genera are *Azoarcus* and *Thauera*. They are followed by *Marinobacter*, *Pseudomonas* and, in some reconstructions, the *Rhodocyclaceae* genera *Azospira* and *Dechloromonas*. The Alphaproteobacteria genera that dominate the P30 culture are remarkably absent in the NP30 culture.

In addition to binning the Metavelvet-assembled contigs by similarity search in global sequence databases, taxonomic composition of the perchlorate-degrading cultures was assessed by cataloging 16S rRNA gene sequences extracted from Metavelvet and Oases assemblies of the metagenomic reads (Additional file 5, Additional file 6). The P30 culture featured 16S rDNA signatures related to the Alphaproteobacteria genus *Roseovarius* (*Rhodobacteraceae*), the Betaproteobacteria genera *Azoarcus*, *Azospira*, *Thauera* and *Rhodocyclus* (*Rhodocyclaceae*), the Gammaproteobacteria genus *Marinobacter* (*Alteromonadaceae*), and several Gammaproteobacteria genera of unclear phylogenetic relatedness (*Sedimenticola*, *Thiobios* and *Thiodictyon*). In the NP30 culture, 16S rDNA sequences belonging to the *Rhodobacteraceae* specifically and the Alphaproteobacteria in general were not observed. The Betaproteobacteria family *Rhodocyclaceae* was represented by all the above-mentioned genera with the addition of the genus *Azonexus* while the list of Gammaproteobacteria was expanded by addition of the genus *Halomonas*. The NP30 culture also exhibited signatures of *Bacteroidetes* and *Spirochaetes*, which were not seen in the P30 culture.

Searches of the metagenomic contigs for genes involved in perchlorate degradation revealed the presence of identical perchlorate reductase gene clusters, *pcrABCD*, and chlorite dismutase genes, *cld*, in both P30 and NP30 cultures. The recovered perchlorate reductase sequence showed highest similarity to an enzyme from *Dechloromonas aromatica* RCB, which is a member of the *Rhodocyclaceae* family. There is 64% sequence identity between the molybdopterin-bearing catalytic subunits (*pcrA*), 67% identity between the iron-sulfur subunits (*pcrB*), and 47% identity between the cytochrome-type subunits (*pcrC*). The closest homolog for the chlorite dismutase found in these cultures is the *cld* gene found in the Betaproteobacterium *Ideonella dechloratans* (47% identity). In view of the negative effect of nitrate addition on the abundance of members of the *Rhodobacteraceae* in the NP30 culture, the metagenomic contigs were searched for genes encoding nitrate-metabolyzing enzymes (Table 3, Additional file 7). It was found that only a few of the functionally annotated P30 and NP30 contigs with highest similarity to nitrate reductase genes are taxonomically affiliated with Alphaproteobacteria taxa including the family *Rhodobacteraceae*. On the other hand, the contigs similar to respiratory and dissimilatory nitrate reductase genes from Beta- and Gammaproteobacterial sources were observed in substantially larger numbers in both P30 and NP30 communities. This observation points to a relatively low nitrate-metabolizing potential of the Alphaproteobacteria fraction of the mixed cultures compared with that of the other taxonomic groups.

**Table 3:** **Taxonomic distribution of metagenomic contigs exhibiting highest similarity to nitrate reductase genes**

**Annotation software**	**MG-RAST**		**MEGAN**		**ContigEval**	
**Similarity search tool**	**Protein BLAT**		**BLASTX**		**BLASTN**	
**Functional annotation source**	**GenBank**		**SEED**		**GenBank**	
	**P30**	**NP30**	**P30**	**NP30**	**P30**	**NP30**
Alphaproteobacteria	5	-	4	1	-	1
Betaproteobacteria	15	16	12	7	28	18
Gammaproteobacteria	12	5	28	12	9	7
Bacilli	1	-	-	-	-	-
Negativicutes	1	-	-	-	-	-
Undefined	-	-	2	1	1	-
Total number of contigs	34	21	46	21	38	26

## Discussion

The stability of microbial populations and function of cultures used for industrial wastewater treatment is an important consideration. It is therefore important to understand what the key organisms are in the P30 and NP30 cultures and ultimately how these populations are effected by changes in the feed brine. The ability to characterize and subsequently monitor culture composition is important for early recognition and treatment of undesirable changes. In this regard, the metagenomic characterization described here is much more informative than earlier methods such as DGGE [21]. Moreover, in light of ongoing advances in high throughput sequencing such as multiplexing and the use of paired end reads, the metagenomics approach is competitive in time and cost potentially allowing routine comprehensive monitoring of the communities at regular intervals in the future.

The communities examined here differ from those used in most other studies of perchlorate and nitrate reducing cultures due to their salt tolerance and longevity. They thus represent unique ecosystems. Comparing the results obtained in this study with the earlier characterization obtained with DGGE [21], it is noticed that the diversity of the cultures has likely decreased with time. Some of the prevailing genera of Gammaproteobacteria, such as *Dechloromarinus* in P30 and *Acholeplasma*-like species of the phylum Firmicutes in both P30 and NP30 observed in 2004 [21] have effectively disappeared in the present study. This change was probably due to the high salt concentrations (6% NaCl) occasionally fed to the two cultures, since similar changes were observed when the cultures were fed with ion-exchange brines [21,22]. The groups of *Rhodocyclaceae* and *Rhodobacteraceae* in P30 had been dominating since 2007 [21] and continue to dominate in this study (sampled in 2010). Members of the Gammaproteobateria (*Marinobacter* and *Halomonas*) observed in the NP30 culture in 2007 [21] are still observed in this study. However, their abundance decreased with members of the Betaproteobacteria (*Azoarcus/Denitromonas*) making up the difference. This may be because of the increased perchlorate concentrations in the NP30 feed that began in 2008. Consistent with this interpretation is the observation that the genus *Denitromonas* emerged to be an abundant member of the NP30 community when the community was fed with only perchlorate for one month [21]. The very defined media fed to the cultures has likely helped to maintain the long-term stability of the cultures, although, the change of the relative ratio of major electron acceptors could greatly change the community. Nevertheless, on the whole, given that the the P30 and NP30 communities are almost exclusively comprised of alpha, beta, and gamma proteobacteria they are far less diverse than what is encountered in natural environments such as soils.

Obtaining key organisms in pure culture will facilitate our understanding of the changes in biochemistry required to effectively utilize perchlorate in the presence or absence of nitrate in saline environments. In addition, such pure cultures could be used in practice to augment the mixed culture should the perchlorate-reducing bacteria be lost or diminished. The metagenomics approach has clarified the likely properties and known relatives of the key organisms in each culture. Thus, to the extent individual organisms can be isolated from the P30 and NP30 cultures, it is now possible to understand their significance in the context of the larger community.

Further effort is needed to obtain a complete set of representative cultures. Previously, a species of *Marinobacter*, a member of the *Alteromonadaceae*, was isolated from the NP30 culture and found to utilize perchlorate in the presence of nitrate in saline environments [24]. The metagenomic results confirm that this organism is likely a significant part of the NP30 community. In the present study, several additional isolates have been obtained from both cultures, but none degraded perchlorate.

An *Azoarcus* like Betaproteobacterium was isolated from the NP30 culture. It belongs to the *Rhodocyclaceae* that dominate the NP30 culture and are highly represented in the P30 culture. On the other hand, *Hyphomonas* sp. was not found in significant numbers in either culture in the metagenomic analysis. However, its distinct cellular morphology allowed it to be observed microscopically as a minor component of both cultures. The other two organisms obtained in pure culture, *Stappia* sp. and *Cellulomonas* sp., do not appear to be present in large amounts in either culture and may have been enriched from small numbers on the solid medium due to their ability to grow in low nutrient conditions. Thus, the isolation of the pure cultures was seemingly influenced by an ability of individual strains to proliferate under selected cultivation conditions in the absence of other members of the microbial communities rather than by their abundance in the mixed cultures.

The *Marinobacter* strain is the only isolate obtained to date that is capable of degrading perchlorate and nitrate simultaneously, although it prefers perchlorate as the electron acceptor [24]. In other studies, a *Marinobacter* species was found as the dominant organism in hydrogenotrophic membrane biofilm reactors (MBfRs) treating ion-exchange brines containing perchlorate [25,26]. Van Ginkel et al. [26] examined the microbial community in four H_2_-based MBfRs treating ion-exchange brines. Four different inocula including NP30 were used in that study. Except the freshwater inoculum, the other three salt-tolerant inocula in the reactors resulted in similar consortia, with members of the Gammaroteobacteria representing 95% and Alphaproteobacteria representing 0-4% of the total 16S rRNA gene clones. Among them, *Marinobacter* constituted 38-81% of the total clones. Even in the freshwater inoculated MBfR, *Marinobacter* represented 34% of the total clones. Since the nitrate fed to NP30 was more concentrated than perchlorate, *Marinobacter* would have a competitive advantage over organisms that could only degrade perchlorate or preferred nitrate to perchlorate. Its significant elevation in numbers in the NP30 cultures relative to the P30 cultures supports this argument. Less clear is why the *Marinobacter* are not present in large numbers in the P30 culture. Although *Marinobacter* can utilize perchlorate under saline conditions the simplest explanation is that the yet to be cultured *Rhodobacteraceae* species are simply better adapted to the saline environment. Previous studies [24] showed that *Marinobacter* sp. P4B1 grew much better at low salt (1.8%) than in the 3% concentration used here.

The genera *Azoarcus* and *Thauera* were consistently found in the metagenome studies as representatives of the *Rhodocyclaceae* that are major components of both cultures*.* These organisms are phylogenetically similar to each other and both are close relatives of the widely-known perchlorate-reducers of the Betaproteobacteria genera *Dechloromonas* and *Azospira* [27]. Members of *Azoarcus* and *Thauera* are known as denitrifiers capable of halobenzoate degradation [28]. In another study, *Azoarcus* was one of the major organisms in a toluene-degrading compost biofilter [29]. In a previous study of the NP30 microbial community by fluorescent in situ hybridization (FISH) [22], *Azoarcus* was proposed to be a major perchlorate reducer in NP30. However, the pure *Azoarcus* strain NY isolated from the NP30 culture in this study was not able to reduce perchlorate after over ~120 days’ incubation. Since nitrate was not available in the P30 mixed culture, the *Azoarcus* strain that was isolated need not be a denitrifier. Two ecologically different groups of *Azoarcus* exist: 1) soil-borne strains which can degrade aromatic hydrocarbons under denitrifying conditions, and 2) “plant-associated” strains which can fix nitrogen and require microaerobic conditions for growth on N_2_ [30]. If strain NY is truly representative of the community, then the members of the *Azoarcus* in P30 and NP30 may be the plant-associated type that are growing under microaerophilic conditions produced during perchlorate degradation.

Matches to *Roseovarius*, *Rhodobacter,* or *Ruegeria* were frequently found in the metagenome studies. Likewise, *Rhodobacter* sequences were consistently observed in DGGE analysis of the P30 culture as well [21]. Unfortunately, no members of the *Rhodobacteraceae* that dominate the P30 cultures have yet been obtained in pure culture. In the course of earlier FISH studies, *Roseobacter* accounted for only 0-5% of the total organisms in the NP30 culture [22]. This is consistent with the much reduced representation of the *Rhodobacteraceae* seen in the metagenome analysis of the NP30 culture described here.

The genus *Stappia* is also in the *Rhodobacteraceae.* Two *Stappia* strains NOW and NYO were isolated in the pure culture studies, but they were recovered unexpectedly from the NP30 culture where *Rhodobacteraceae* are present in small numbers. The genus was created from reclassification of marine *Agrobacterium* [31] and is closely related to *Ruegeria*. They usually form star-shaped-aggregates [32], which were observed in the isolated *Stappia* strain NW. The type species of *Ruegeria*, *Ruegeria atlantica*, could reduce nitrate to nitrite and to N_2_ gas [32], while some species of *Ruegeria* could only reduce nitrate to nitrite but not to N_2_ gas [31]. Neither of the pure strains, NWO and NYO, could reduce perchlorate.

Previous attempts to use real time PCR and other biochemical techniques from the literature to quantify and or isolate and purify enzymes associated with perchlorate and/or nitrate reduction were unsuccessful. The metagenome data allowed a preliminary search for such genes. Many, e.g. the perchlorate reductase genes belonging to the *pcrABCD* cluster and the chorite dismutase genes, were in fact found in both the P30 and NP30 cultures. However, the matches to known sequences were moderate to low, which likely explains earlier difficulty in amplifying them. This difference may be due to the saline environment.

## Conclusions

The major components of the P30 and NP30 microbial communities have been established and several representative strains obtained in pure culture. Members of the *Rhodobacteraceae* dominate the P30 culture, but their numbers dramatically decrease in the NP-30 culture that is fed both nitrate and perchlorate. The *Rhodobacteraceae* are largely replaced in the NP30 culture with members of the *Rhodocyclaceae* and to a lesser extent the *Altermonadaceae*. Consistent with their common ancestry, the rest of the community remains similar in the two cultures. With the community structure established at the Family level, it will now be possible to attempt to culture additional key community members by utilizing published recipes and growth conditions of known members of the same families.

Although almost all known perchlorate-reducing organisms can effectively reduce nitrate, the *Rhodobacteraceae* that dominate the P30 culture apparently can’t and thus may represent a novel group of perchlorate reducers. These *Rhodobacteraceae,* as well as other organisms in these cultures, may be a promising source of unique salt-tolerant enzymes for perchlorate reduction.

## Methods

### Maintenance of P30 and NP30 cultures

The cultures were continuously maintained in the laboratory by spiking the electron acceptor and donor once a day along with weekly medium replacements using SBR procedures as described previously [22]. This provided a concentration in the culture of 100 mg/L sodium perchlorate (P30) or 100 mg/L perchlorate and 500 mg/L sodium nitrate (NP30). Beginning in 2008, the perchlorate fed to NP30 was increased to 500 mg/L. The cultures typically removed nitrate within 3–4 hours and perchlorate within 8 hours. The biomass concentration varied due to use as inoculum for experiments, but was typically 2000 mg/L as VSS.

### Pure culture isolation

The selective agar medium for isolation of the pure cultures contained 0.62 g/L NaClO_4_, 30 g/L NaCl, and 12.5 g/L agar. The basal perchlorate (P) medium for cultivation contained 0.62 g/L NaClO_4_, 1.24 g/L CH_3_COONa · 3H_2_O, 0.25 g/L yeast extract, and 0.25 g/L peptone, 11 g/L MgCl_2_ · 6H_2_O, 1.4 g/L CaCl_2_ · 2H_2_O, 0.72 g/L KCl, 30 g/L NaCl, 0.3 mL/L resazurin (0.1%), 200 mg/L NaHCO_3_ (*), 5.0 mL/L of 67 mM Na_2_S · 9H_2_O (*), 1.0 mL/L of 50 g/L KH_2_PO_4_, and 1.0 mL/L of mineral solution. The asterisk (*) indicates items added to the media after boiling and cooling to room temperature. The mineral solution contained: 10 g (NH_4_)_6_Mo_7_O_24_ · 4H_2_O, 0.05 g ZnCl_2_, 0.3 g H_3_BO_3_, 1.5 g FeCl_2_ · 4H_2_O, 10 g CoCl_2_ · 6H_2_O, 0.03 g MnCl_2_ · 6H_2_O, and 0.03 g NiCl_2_ · 6H_2_O in 1 L deionized water.

The basal nitrate (N) medium was identical to the P medium except that 0.62 g/L NaClO_4_ was replaced by 0.68 g/L NaNO_3_. All media were sterilized by autoclaving at 121°C for 30 min. Nitrate and perchlorate were analyzed with an ion chromatograph (ICS-3000, Dionex, Sunnyvale, CA) equipped with AS16 and AS20 columns using dual system chromatography. The detection limits for NO_3_^−^ and ClO_4_^−^ of this method were 10 μg/L and 1 μg/L, respectively.

Serial dilutions of the mixed cultures NP30 and P30 were streaked on selective agar plates and incubated in an anaerobic glove box maintained under oxygen-free atmosphere (80% N_2_, 10% CO_2_ and 10% H_2_). When single colonies appeared, they were picked, streaked on fresh plates and incubated again either in the anaerobic glove box or in the air. After 5 days’ incubation, colonies were picked off the plates and transferred into Hungate tubes containing 10 mL P or N medium. In total, eight isolates were obtained. The growth of these isolates in the presence of perchlorate, nitrate and oxygen was examined using the measurement of optical density at 520 nm (OD_520_).

### DNA extraction, preparation and sequencing

1. DNA isolation

The Promega Wizard® Genomic DNA Purification kit (Promega Corp. Madison, WI) was used to extract the DNA from the cultures from 1 mL culture as per the directions with the kit. The DNA was stored at 4°C prior to use.

(2) 16S rDNA Sequencing

Fragments of the 16S rRNA gene were amplified by PCR from cellular DNA obtained from each pure culture using 16S rDNA-specific primers 27 F, 5’-AGAGTTTGATCMTGGCTCAG [33], and U1510R, 5’-GGTTACCTTGTTACGACTT [34], purchased from Eurofins MWG (Huntsville, AL). The reaction was performed with 20 units/ml *Taq* DNA polymerase in 1 × ThermoPol buffer (New England Biolabs, Ipswich, MA) supplemented with 0.2 mM of each dNTP and 5 μM of forward and reverse primers. The PCR conditions were as follows: initial denaturation at 94°C for 5 min, 32 cycles consisting of denaturation at 94°C for 0.5 min, annealing at 52°C for 0.5 min, and extension at 72°C for 1 min, and final elongation at 72°C for 10 min. PCR products were column-purified [35] and sequenced bi-directionally by the dye-terminator method with the same primers used for amplification. Sequencing was performed by SeqWright, Inc. (Houston, TX).

(3) Metagenome Sequencing

DNA samples for shotgun sequencing were prepared according to the protocols provided by Illumina Inc. (San Diego, CA). In brief, purified metagenomic DNA was nebulized to produce fragments of less than 800 bp. The resulting oligonucleotide mixtures were end-repaired, A-tailed and converted to dsDNA libraries tagged with Illumina adapters for single end sequencing. The libraries were amplified, gel purified, and finally dissolved in water. Quantification of the libraries was done using PicoGreen fluorimetric assay [36]. Sequencing was performed on Illumina Genome Analyzer IIx System according to the manufacturer’s specifications.

### Data analysis

1. 16S rRNA

The closest relatives of the partial 16S rRNA gene sequences were identified in the NCBI *nt* database (ftp://ftp.ncbi.nih.gov/blast/db) using BLAST 2.2.27 + [37]. Taxonomic affiliations were derived from the best hits excluding matches to uncultured or unidentified organisms.

(2) Metagenome Analysis

**Figure 3:**
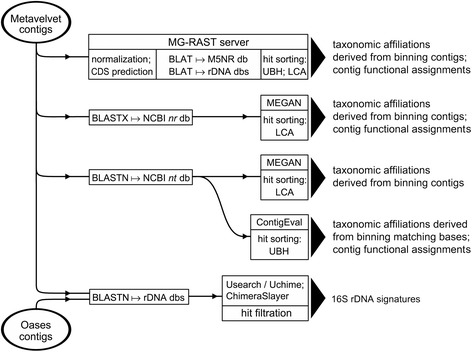
**Flowchart of metagenome data analysis.** The assembled contigs were evaluated against global sequence databases (NCBI *nt* and *nr*, MG-RAST M5NR) and rDNA databases (Silva, Greengenes, RDP, NCBI *16SMicrobial*). Taxonomic binning was performed using either Unique Best Hit (UBH) or Lowest Common Ancestor (LCA) algorithm.

The overall strategy of metagenomic datasets analysis is presented on Figure 3. The quality of the collected sequencing reads was assessed with the fastQC 0.10.1 tool (http://www.bioinformatics.babraham.ac.uk/projects/fastqc). The reads were processed with the FASTX toolkit (http://hannonlab.cshl.edu/fastx_toolkit) to remove terminal low-quality nucleotides, and then assembled using Metavelvet 1.2.02 which is an extension of the Velvet assembler designed to do *de novo* metagenome assembly from short sequence reads [38]. It is bundled with Velvet 1.2.08 [39]. The *k*-mer length and the minimum contig size were set to 19 and 100, respectively. The assembled contigs were submitted to the MG-RAST 3.3 server [40] for taxonomic assignment and functional annotation. Alternatively, the contigs were evaluated batchwise using BLASTN searches against the Silva rRNA database (version 111) [41] and/or the NCBI *nt* and *16SMicrobial* databases (ftp://ftp.ncbi.nih.gov/blast/db). BLASTX searches against the NCBI *nr* database (ftp://ftp.ncbi.nih.gov/blast/db) were also conducted. Primary sorting of BLAST search results was performed using BlastParser 1.2 (http://geneproject.altervista.org). Contigs that matched database entries with E-value < 10^−10^ (NCBI *nt* and *nr* databases) or < 10^−5^ (NCBI *16SMicrobial* and Silva rRNA databases) were selected for further analysis. Taxonomic classification of BLASTX hits was performed with MEGAN 4.70.4 [42] using the lowest common ancestor (LCA) algorithm. The same approach was used to evaluate BLASTN matches with the Silva rRNA and NCBI *16SMicrobial* databases. Annotation of BLASTN hits in NCBI *nt* database was performed using proprietary Perl scripts (ContigEval pipeline) for sorting the BLAST output, retrieval of taxonomic information from NCBI *nodes.dmp* and *names.dmp* files (ftp://ftp.ncbi.nih.gov/pub/taxonomy), and identification of the hit genes on subject nucleotide sequences by parsing NCBI *gene2accession* and *gene_info* files (ftp://ftp.ncbi.nlm.nih.gov/gene/DATA). Contigs mapped to sequences of particular interest were further extended using targeted assembly software, Mapsembler 1.3.16 [43]. In addition, some contig lengthening was achieved by running a transcriptome assembler, Oases 0.2.08 [44] in metagenomic context with *k*-mers ranging from 19 to 31 and coverage cut-off set to 3. Oases was used only in the reconstruction of rRNA and perchlorate reductase gene sequences. Due to the significant probability of chimeric assembly, the Oasis-produced contigs were extensively filtered using the Uchime tool from the Usearch 6.0 software package [45] and ChimeraSlayer release 20110519 [46].

(3) GenBank Accession Numbers

Partial 16S rRNA gene sequences of isolated strains NWO, NY, NYO, PW, PWO, PY and PYO were deposited in GenBank under accession numbers KF135667-KF135673, respectively. Nucleotide sequences encompassing *pcrABCD* gene clusters recovered from NP30 and P30 mixed cultures are assigned accession numbers KF135674 and KF135675, respectively. Chlorite dismutase (*cld*) gene sequences from NP30 and P30 mixed cultures are placed under accession numbers KF135676 and KF135677,

## Additional files

Additional file 1:
**Taxonomic composition of P30 and NP30 microbial communities estimated using MG-RAST server.**
Additional file 2:
**Taxonomic composition of P30 and NP30 microbial communities estimated using MEGAN program.**
Additional file 3:
**Taxonomic composition of P30 and NP30 microbial communities estimated using ContigEval pipeline.**
Additional file 4:
**Relative abundance of major bacterial families in P30 and NP30 communities shown ordered by taxon abundance averaged for P30 and NP30 cultures.**
Additional file 5:
**Identification and taxonomic assignment of 16S rRNA gene sequences in P30 and NP30 metagenomic contigs.**
Additional file 6:
**16S rDNA signature sequences identified in P30 and NP30 metagenomic contigs.**
Additional file 7:
**Identification and taxonomic assignment of nitrate reductase gene sequences in P30 and NP30 metagenomic contigs.**
