# Supplementary material for: The presence of nitrate dramatically changed the predominant microbial community in perchlorate degrading cultures under saline conditions
Source: BMC Microbiol. 2014 Sep 7;14:225. doi: 10.1186/s12866-014-0225-3 (PMC4174249; doi:10.1186/s12866-014-0225-3)
Supplement: Additional file 4: — Relative abundance of major bacterial families in P30 and NP30 communities shown ordered by taxon abundance averaged for P30 and NP30 cultures. [file 12866_2014_225_MOESM4_ESM.pdf]

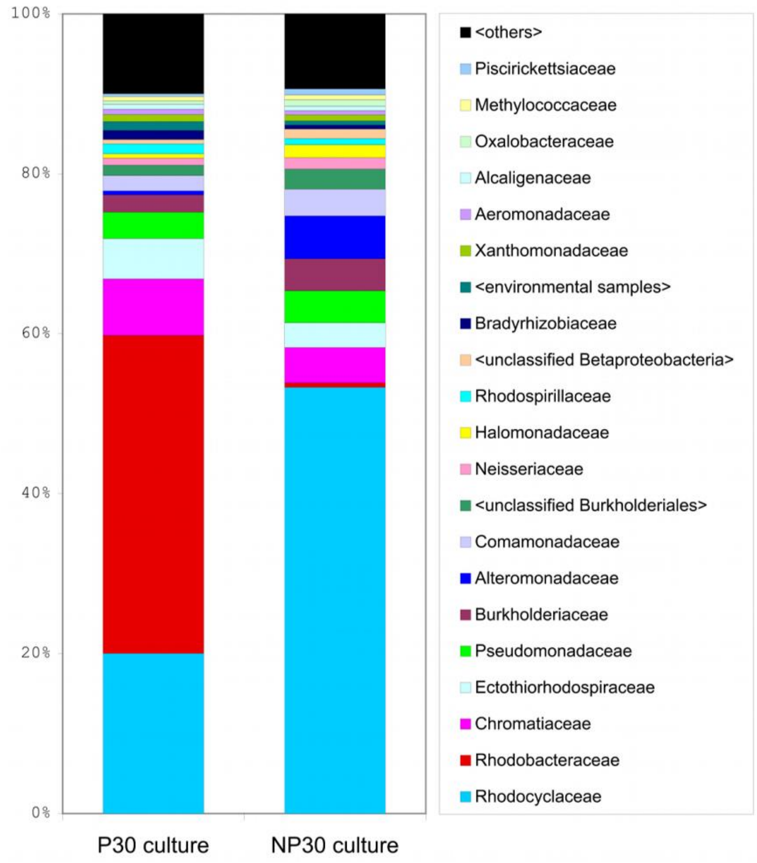


**Figure 2.** Relative abundance of major bacterial families in P30 and NP30 communities estimated using ContigEval analysis pipeline. Height of the colored boxes represents a number of identities in all contigs assigned to a given taxon related to a number of identities in all contigs assigned to all taxa. The boxes are ordered by a taxon abundance averaged for P30 and NP30 cultures. For each culture, only the most abundant families contributing to the 90 % of cumulative percentage are shown.
